# *In Vitro* Blood-Brain Barrier Models for Neuroinfectious Diseases: A Narrative Review

**DOI:** 10.2174/1570159X22666231207114346

**Published:** 2023-12-08

**Authors:** Ahmad Hussein Badawi, Nur Afiqah Mohamad, Johnson Stanslas, Brian Patrick Kirby, Vasantha Kumari Neela, Rajesh Ramasamy, Hamidon Basri

**Affiliations:** 1 Department of Neurology, Faculty of Medicine and Health Sciences, Universiti Putra Malaysia, 43400, Serdang, Selangor, Malaysia;; 2 Centre for Foundation Studies, Lincoln University College, 47301, Petaling Jaya, Selangor, Malaysia;; 3 Department of Medicine, Faculty of Medicine and Health Sciences, Universiti Putra Malaysia, 43400, Serdang, Selangor, Malaysia;; 4 School of Pharmacy and Biomolecular Sciences, RCSI University of Medicine and Health Sciences, Dublin, Ireland;; 5 Department of Medical Microbiology and Parasitology, Faculty of Medicine and Health Sciences, Universiti Putra Malaysia, 43400, Serdang, Selangor, Malaysia;; 6 Department of Pathology, Faculty of Medicine and Health Sciences, Universiti Putra Malaysia, 43400, Serdang, Selangor, Malaysia

**Keywords:** Blood-brain barrier (BBB) model, standardization of BBB model, optimization parameters, validation parameters, neuroinfectious diseases, peripheral blood system, central nervous system

## Abstract

The blood-brain barrier (BBB) is a complex, dynamic, and adaptable barrier between the peripheral blood system and the central nervous system. While this barrier protects the brain and spinal cord from inflammation and infection, it prevents most drugs from reaching the brain tissue. With the expanding interest in the pathophysiology of BBB, the development of *in vitro* BBB models has dramatically evolved. However, due to the lack of a standard model, a range of experimental protocols, BBB-phenotype markers, and permeability flux markers was utilized to construct *in vitro* BBB models. Several neuroinfectious diseases are associated with BBB dysfunction. To conduct neuroinfectious disease research effectively, there stems a need to design representative *in vitro* human BBB models that mimic the BBB's functional and molecular properties. The highest necessity is for an *in vitro* standardised BBB model that accurately represents all the complexities of an intact brain barrier. Thus, this in-depth review aims to describe the optimization and validation parameters for building BBB models and to discuss previous research on neuroinfectious diseases that have utilized *in vitro* BBB models. The findings in this review may serve as a basis for more efficient optimisation, validation, and maintenance of a structurally- and functionally intact BBB model, particularly for future studies on neuroinfectious diseases.

## 1 INTRODUCTION

The blood-brain barrier (BBB) is a selective structural and functional barrier that protects the central nervous system (CNS) from unrestricted access to molecules from circulating blood, which is required for the physiological functionality of glial cells and neurons. The BBB also stabilises the neural microenvironment by limiting the infiltration of the CNS and circulating neurotoxic agents, inflammatory factors, immune cells, or pathogens [1-4]. A disruption in the BBB may be due to the redistribution or degradation of tight junction proteins (TJP), causing enhanced BBB permeability [5, 6]. The recruitment of leukocytes into the brain is a prominent pathological feature in many neuroinfectious diseases,including cerebral infection [7] and multiple sclerosis [8]. This results in durable neurologic deficits and high mortality and morbidity rates globally [9].

Multidrug resistance proteins, such as P-glycoprotein, obstruct drug transport across the BBB that might promote the development of “sanctuary sites”, permitting pathogens to replicate in treated patients' brain tissue [10]. Despite advances in vaccines and antibiotic therapy, several microbial infections continue to be significant public health problems, resulting in a high rate of mortality and morbidity. Therefore, understanding the mechanisms by which pathogens penetrate the BBB is essential for future rapid diagnosis and treatment of neuroinfectious diseases. Animal models can contribute significantly to our understanding of the pathophysiology of neurodegenerative diseases. However, study findings through animal models have limited applicability to the human BBB as animal models have a higher transendothelial electrical resistance (TEER) value than human BBB models [11, 12]. There are also considerable species-specific differences in both animal and human BBB, including the timing of BBB maturation, clearance rates of amyloid-beta, different levels of expression and functions of BBB transporters, and differences in the activities of BBB metabolic enzyme [13]. Therefore, increased research on human BBB *in vitro* models has permitted a more detailed experimental study of the BBB, mechanical knowledge of the brain endothelium-pathogen interaction, an overview of human BBB pathophysiology, and leading to reduced dependence on animal models.

In this review, we present an overview of *in vitro* BBB models that are currently being utilised to study neuroinfectious diseases. We describe the various types of cells that compose the BBB, the factors that influence BBB permeability, the optimization and validation of parameters for a BBB model, and the applications of *in vitro* BBB models in previously published studies.

## 2 HUMAN BLOOD-BRAIN BARRIER CELLS AND BLOOD-BRAIN BARRIER PERMEABILITY

The BBB is a complex structure composed of diverse multicellular components that form a functional “neovascular unit”, which includes human brain endothelial cells that line the cerebral vasculature [14], capillary basement membrane, brain pericytes embedded within the basement membrane [15], and end-feet of brain astrocyte ensheathing the vessels [16-18].

Brain microvascular endothelial cells (BMECs) have specialised transport systems, a uniform thickness without transendothelial fenestrations, a low level of pinocytotic activity, continuous intercellular tight junctions, and a high mitochondrial volume [19, 20]. The existence of continuous tight and adherens junctions in the BMECs almost completely seals the paracellular space between adjacent lateral endothelial membranes [21-25].

Pericytes are critical capillary and post-capillary venule cellular constituents. They share the same basement membrane as endothelial cells [26] and cover 22-32% of capillaries in the CNS [27]. Pericytes are involved in various neurovascular functions, including angiogenesis, BBB formation during embryogenesis, vascular stability maintenance, capillary blood flow regulation, and eliminating toxic cellular products [28]. Pericytes can influence the production of tight junction molecules in endothelial cells *via* secreting factors, for instance, angiopoietin [29] and TGF-α [17]. TGF-improves BBB function by inhibiting leukocyte migration and endothelial cell proliferation. The release of angiopoietin can result in capillary remodelling and stabilisation. Endothelial cells secrete platelet-derived growth factor (PDGF), which is required to recruit and maintain pericytes on vessels and vascular maturation [30, 31].

Astrocytic end-feet cover 99% of the microvessel surface in the brain, with only a thin basal membrane separating their end-foot processes [18]. Astrocytes regulate numerous physiologic aspects of the BBB through the action of critical regulatory factors such as transforming growth factor-β (TGF-β) [17], glial-derived neurotrophic factor (GDNF) [32], and the fibroblast growth factor (FGF) [33]. Additionally, astrocytes affect specific BBB characteristics such as permeability, tight junction formation, and endothelial cell expression [34].

Neurons are typically 8-20 mm away from brain capillaries [35]. Neurons are also actively involved in forming BBB structure, as neuronal terminations are present in all BBB-forming cells [36, 37]. Neurons remain close to capillaries and link to astrocytic end-feet near the BBB. Due to their proximity to endothelial cells, neurons are able to respond to the constantly shifting local environment (*e.g*. ion balance). Neurons play a function in controlling blood flow, microvascular permeability, extracellular matrix interactions, and the release of substances that drive angiogenesis [38]. Signals from neurons and astrocytes can recruit microglia that produce proinflammatory cytokines in response to a vascular insult [39]. Neurons can induce and sustain BBB characteristics *in vitro* [40, 41]. Neurons contribute to the contraction of brain endothelial cells in culture by facilitating the synthesis and localization of tight junction proteins [41]. The production and function of BBB efflux transporters are regulated by neuronal activity, which is essential for excluding numerous tiny lipophilic compounds from the brain parenchyma. In addition, in brain endothelial cells, neuronal activity affects the expression of circadian clock genes, subsequently driving the activity-dependent regulation of BBB efflux transport [42]. These findings support a synergistic function for neuronal regulation of different cell types and emphasise the intercellular communication between these cells. Water channels within astrocytes connect the neural circuitry to the blood arteries [43].

Perivascular macrophages/microglia are the resident immune cells of the CNS. This cell holds a critical position in the BBB, allowing for the modulation of the brain's innate and adaptive immune responses. Human brain perivascular microglia express molecules implicated in antigen recognition, antigen presentation, and co-stimulation [44]. Therefore, microglia are an additional line of immune defence against infections or toxins that cross the BBB. Their function involves regulating BBB inflammation, maintaining endothelial cell health, stability and integrity of the BBB, regulation of vasoconstriction, and participation in angiogenesis and BBB recovery (TJP delivery) [45, 46]. Table **1** further summarises the characteristics and functions of human BBB cells.

Several factors and indicators determine the permeability of the BBB from inter-endothelial junctions, transporters and molecular markers. Adherens junctions induce endothelial cell adhesion, contact inhibition during vascular growth and remodelling, initiate cell polarity, and regulate paracellular permeability. Another inter-endothelial junction, the tight junction, acts as a physical barrier, forcing most molecules to cross the BBB *via* a transcellular pathway rather than a paracellular pathway. Both efflux and solute-like carrier (SLC) transporters are expressed on brain endothelial cells, which play crucial roles in the permeability of small molecules across the BBB. Their expression profile largely dictates their permeability properties and functions, which is a critical prerequisite for an *in vitro* BBB model [99, 100].

It is well established that the BBB is relatively impermeable under normal physiological conditions due to the presence of inter-endothelial junctions and transporters that regulate the pathways of substances. However, during pathologic conditions, multiple sources have been demonstrated to release chemical mediators, vasoactive agents, and cytokines/chemokines into tissues and the bloodstream, thereby disrupting the BBB’s permeability. Previous *in vivo* and *in vitro* studies have reported that several mediators, such as ATP, aspartate, glutamate, taurine, nitric oxide (NO), endothelin-1, TNF-α, and macrophage-inflammatory protein 2 (MIP2) produced by astrocytes, vasoactive agents (free radicals, 5HT, bradykinin, histamine, thrombin, substance P, quinolinic acid, UMP, UTP, and platelet-activating factor) and free radicals, interleukin-1 (IL-1) and interleukin-6 (IL-6) generated by bacterial protein lipopolysaccharide (LPS) could alter the BBB permeability [14, 47, 99-103].

Another significant regulator that affects the barrier’s permeability during inflammation is the vascular endothelial growth factor A (VEGFA) and thymidine phosphorylase (TYMP), which act synergistically to suppress the expression of occludin and claudin-5, which acts synergistically to suppress the occludin and claudin-5 expression, two transmembrane proteins required for the integrity of tight endothelial junctions [104, 105]. It has been demonstrated that the astrocyte-derived VEGFA is a significant contributor to barrier dysfunction *via* endothelial nitric oxide synthase (eNOS), which inhibits claudin-5 and occludin expression [106, 107]. Furthermore, along with the endothelial barrier antigen (EBA) and immunoglobulin G (IgG), VE-cadherin is a cell adhesion molecule expressed by the vascular endothelium [108]. It is mainly found at cellular junctions [109, 110] and is utilised as an indicator of the BBB’s loss of integrity [111]. Another factor that could play a significant role in disrupting the BBB’s permeability is the attenuation in caveolin-1 and MMPs levels [107, 112] since caveolin-1 is the caveolae's primary structural protein and may contribute to vesicular trafficking and cell signalling [113].

Several neuroinflammatory models [105] have reported the association of BBB permeability disruption with proinflammatory cytokines and chemokines response [114, 115]. For instance, The C-C motif chemokine ligand 2 (CCL2) disrupted the BBB barrier by binding to C-C chemokine receptor type 2 (CCR2) on the brain endothelium, thus disrupting the TJP [116, 117]. CCL2 is a crucial chemokine that mediates immune cell adhesion to the cerebral endothelium [118]. It is secreted by astrocytes and BMECs at the glia limitans, facilitating immune cell immunosurveillance activity within the BBB vasculature [119, 120].

## 3 *IN VITRO* BLOOD-BRAIN BARRIER MODEL

To accurately mimic a BBB condition, an *in vitro* BBB model must exhibit the same morphological, physical, and functional characteristics as the *in vivo* model. The BBB cells should be derived from human sources and preserved in a physiological and morphological environment consistent with that of the BBB. Co-culture of brain endothelial cells and other BBB cellular and structural components is recommended, and shear stress should be incorporated into the BBB model to mimic the in *vivo* blood flow. Additionally, the model should permit inflammatory cells to migrate transendothelial, which can be used to perform additional analyses such as cell counting, cellular markers, and viability. Finally, the model should allow for the incorporation of cytokines or chemokines to study their effect and role [121].

### 3.1 Types of *in vitro* Blood-brain Barrier Models

Many models of BBB have been proposed, including monoculture, co-cultures, triple cultures, quadruple cultures, and 3D ECM-based Transwell models and dynamic systems, including dynamic *in vitro* BBB model (DIV-BBB), microfluidic BBB (μBBB), and BBB-on-a-chip.

The benefits and drawbacks of these systems have been extensively explored in previous literature; thus, the advantages and disadvantages of different *in vitro* BBB models are summarized in Table **2**. Validation markers, including the expression of BBB enzymes, transporters, receptors, and structural proteins, are utilised to generate overviews of the currently existing models for comparison and model selection [122-127].

### 3.2 Three-dimensional *in vitro* Blood-brain Barrier Models

Three-dimensional models (3D) are a crucial step forward in BBB modelling. Establishing the BBB in artificial microvessels is accomplished by the growth of BEC in the coated channels lumen to build a microstructure with the addition of perivascular cells (pericytes and astrocytes) on the outer surface of the channels [123]. In the specific example (3D ECM-based BBB models), brain microcapillaries are grown on self-polymerizing ECM scaffolds where the BBB cellular components can form tight contacts while being exposed to trophic nutrients along quasi-physiological biochemical gradients [124].

The advantage of the 3D model is: (a) the topographic distribution of BBB’s cellular component, which mimics the *in vivo* BBB, (b) the barrier characteristics resemble those *in vivo* more closely (transporter expression, cell polarization, and high TEER), and (c) a more realistic microcirculation environment comprising real-time monitoring, an oxygen permeable substrate, presence of shear stress and fluid flow, and biological measurement [123]. This design permits a more physiologically appropriate geometry and cell-cell interaction for investigating permeability and inflammatory response [128].

Due to the lack of flow and limited media exchange in two-dimensional (2D) models, the glucose consumption and lactate production of BEC are high [129]. This could convert the cellular metabolism to anaerobic metabolic pathways in pre-experimental circumstances and influence the phenotypic and pathological response of BEC.

Two-dimensional models are reproducible and cost-effective systems for drug transport and cell migration studies [130]. DIV-BBB offers a solid foundation for comprehending the influence of shear stress on BECs under diverse pathological states [130]. Potential applications of 3D models (μBBB, BBB-on-chip) include pharmacology (drug absorption, dosage, and drug development), disease-target research, and translational medicine [131].

### 3.3 Gold Standard or Ideal Model

There is currently no ideal or gold standard *in vitro* BBB model being developed due to a limited understanding of the human BBB, which makes determining the characteristics of an ideal *in vitro* BBB model challenging. On the other hand, an ideal model should meet several critical parameters, including the structural and functional features outlined in Table **3**. The *in vitro* BBB model should be able to mimic the effects of different haemodynamic and immune/inflammatory insults on the BBB that may contribute to the aetiology and progression of CNS diseases [132].

A perfect *in vitro* BBB model should satisfy several essential criteria (Table **3**). These include (a) enabling interendothelial tight junction expression between adjacent ECs, hence facilitating the creation of an extremely strict and selective BBB barrier (limited paracellular diffusion), (b) *in vivo*-like asymmetric-distribution (apical *versus* basolateral) of necessary transporters [133] which confers ECs polarisation, (c) the expression of functioning drug efflux and drug metabolism pathways [134-136], and (d) the capacity to distinguish the permeability of different substances [137].

The development of realistic *in vitro* BBB models that mimic the physiological and molecular properties of the NVU/BBB is crucial for CNS drug discovery, neuroinfectious research, and translational medicine. Successful modelling of the NVU would help dissect pathogenic causes and mechanisms of action (and targets) preceding the onset of CNS diseases. It is claimed that inadequate BBB model selection may affect data reliability and cause suboptimal clinical trial outcomes [122, 132].

#### 3.3.1 Differences between Primary versus Immortalized Cell Lines of BBB and Animal versus Human-derived Cells

In previous work, animal cells rather than human cells were often used in *in vitro* models. Porcine or murine brain endothelium and rat astrocytes are employed in models because they are more accessible and economical to maintain, and well-characterized [138, 139]. These models are cultivated using an animal serum. How effectively can non-human models be extended to humans *in vivo*? Fully human models are needed to evaluate non-human models. Human brain-derived cells and other cell types cultured in serum-free environments, foetal bovine serum, and human serum show antigens and gene expression changes, which may cause junction tightening [140]. Animal and human serum can change cell morphology and tight junction development [141].

The differential expression of BBB transporters levels also indicates that there are some differences between the functions of the BBB in animals and humans [142-144]. P-gp expression differs between the rat and human models, presumably due to the fact that P-gp is encoded by two genes in rats but only one gene in humans [145]. Consequently, their regulation and protein expression may change.

Endothelial cells from diverse sources have been utilised to model *in vitro* BBB, including PSC-derived, primary and immortalised cells across various mammalian species. Primary BMECs are challenging to purify and rapidly lose their *in vitro* BBB phenotype [146], whereas immortalised BMECs are more convenient but frequently exhibit weak barrier functionality [147]. Other NVU cell types may have similar limitations due to their primary or immortalised cell sources, typically derived from animal tissues.

The cell type utilised is the most crucial aspect of any *in vitro* NVU/BBB model. This model's usability, cost-effectiveness, and translational relevance are all dependent on the cells utilised to build it *in vitro* since a more accurate representation of the physiological properties and response of the BBB can be achieved by this method. In addition, these cell types' availability and expandability can considerably impact the cost of operating and maintaining the platform [148].

Models of BBB derived from primary cells are the most accurate. Their relatively good correlations with *in vivo* models are typically associated with relatively high TEER values and low paracellular permeability of trace markers [149]. A disadvantage of these models is that the expression of transporter proteins and efflux pumps differs between species [126, 128], which has been proven to be a restrictive barrier of animal brain-derived primary endothelium cells *in vitro* [64, 150, 151]. Models employing primary cultures of human origin would eliminate species-specific differences; however, the availability of such cells is limited for ethical reasons.

The limitations of primary human cells include (a) primary cells are limited and often require a clinical equivalent from whom to extract brain tissue, (b) Time-consuming and low-yield [148], (c) primary cells are prone to contamination by NVU cell types such as astrocytes and pericytes, which can bring confounding variables such as altered cell monolayer layout and unintentional induction of BBB features to ECs [152], and (d) isolation and purification demand technical skills. Cells obtained from human brain tissue excision are likely related to the underlying brain disease. *In vitro*, these cells are more likely to have pathogenic features (such as drug resistance) [153, 154]. This is a benefit not afforded by cell lines or animal-derived primary cells; however, these cells are limited in availability [122], donor-to-donor variable, and disease-specific differences. Cell survival is a concern since primordial cells differentiate quickly in culture and dedifferentiate spontaneously after repeated passages. *In vitro* cell viability-carrying properties are also important, especially for human cells [122].

Due to these limitations, cerebral capillary endothelial cells have been immortalised with human telomerase reverse transcriptase catalytic subunit and Simian vacuolating virus 40 (hTERT/SV40) Large T antigen [147]. However, existing human brain capillary endothelial cell lines frequently exhibit defects, such as low TEER values, relatively high paracellular penetration of negative control substances, and inadequate expression of essential transporter systems [11]. Several factors, including cell types and co-culture media, should be considered when developing an optimal BBB *in vitro* model.

### 3.4 Optimization Factors of an *in vitro* Blood-brain Barrier Model

New cell culture support materials (anchoring/adhesive molecules), such as a matrix structure containing the desired anchoring or adhesive molecules (for cell adhesion or to elicit specific cellular responses), would allow researchers to more finely control cell differentiation, cellular interaction, and cellular response [132]. A larger surface area and the type of coated proteins, such as a fibronectin and collagen IV mixture coating, may significantly influence the endothelial basement membrane compared to a membrane/insert pre-coated with collagen. The culture period is also a factor that facilitates the proper formation of inter-endothelial adherents and tight junctions [155]. Other factors, such as initial cell loading, real-time monitoring of TEER values, and medium sampling, are also optimizable factors of an *in vitro* BBB model, which are further summarised in Table **4**.

### 3.5 Validation of *in vitro* Blood-brain Barrier Model

Several methods of validating an *in vitro* BBB model are available. One frequently used technique is determining the TEER values arising from the cell-cell interaction of endothelial cells cultivated on a porous membrane. TEER is generally expressed as measured resistance (ohm) multiplied by the surface area of the endothelial monolayer (cm^2^). The membrane surface varies considerably and could have a value of 0.3 cm^2^ (24-well plate), 1 cm^2^ (12-well plate), and 4.2 cm^2^ (6-well plate). A higher TEER of the monolayer cells reflects a tighter barrier. Consequently, it is recommended to indicate the surface area of inserts in published studies [11]. Setting an ideal TEER value for a fully functional BBB model is challenging due to the wide variety of TEER values reported in numerous research studies using different models.

TEER values have been commonly used to evaluate the functionality of a BBB model in various species [156]. For instance, the TEER physiological resistance of the frog brain microvascular endothelium was reported to be greater than 1000 Ω.cm^2^ [157]. In contrast, the TEER of the BBB in rats was measured at 5900 Ω.cm^2^, which was considered relatively high compared to the available *in vitro* models [158]. The endothelial electrical resistance of the tight junctions between BBB endothelial cells *in vivo* ranges between 1500 Ω.cm^2^ and 2000 Ω.cm^2^ (pial vessels) [158, 159]. In light of this, endothelial cells are co-cultured with other brain cells in *in vitro* models to increase TEER values and replicate *in vivo* models [156].

The permeability of endothelial monolayer to tracer compounds with defined molecular weight, such as sodium fluorescein or fluorescein isothiocyanate (FITC)-dextran, Evans blue, and horseradish peroxidase, was recommended for the validation of an *in vitro* BBB model [180]. Monolayer cells need to be evaluated *via* uncharged solutes with differing sizes, which are highly inert and non-toxic and are not taken up by the cells. Choosing a range of tracers is essential in comprehensively evaluating monolayer integrity [181]. In general, because of the existence of tight junctions, the exogenous tracers would not be able to diffuse into the brain parenchyma unless the BBB permeability has been disrupted. As a result, they are a valuable tool for validating an *in vitro* BBB model [180].

Additionally, gene and protein expression analyses are performed to evaluate the structure and function of *in vitro* BBB models [182]. It is critical to investigate the changes in the expression of permeability-related genes because gaps in monolayer cells caused by improper handling or cell seeding, as well as changes in the ionic composition of the growth medium induced with specific treatments, could potentially lead to errors in the permeability measurement. Expression analyses also allow comparisons between the BBB integrity and gene expression of BBB-related proteins [183]. Improving *in vitro* BBB models is a principal challenge [182] and requires proper validation of various parameters, as summarised in Table **5**. Meanwhile, the advantages and limitations of each method used to optimize and validate the *in vitro* BBB models are presented in Table **5**.

### 3.6 Maintenance of *in vitro* Blood-brain Barrier Model

The features of the BBB are maintained by interactions between BBB cells (brain microvascular endothelial cells, pericytes, astrocytes, neurons, and microglia) [16, 196]. Abnormal interactions among these cells may interrupt the barrier function. Therefore, the maintenance of BBB structural and functional properties, including maintaining cell viability, constructing the tissue scaffold itself, monitoring cells for dedifferentiation after reaching complete differentiation, and confirmation of maintenance of BBB integrity, is required [162].

### 3.7 Designing Inflammatory *in vitro* Blood-brain Barrier Models

Neuroinflammatory substances are capable of impairing the tight junction integrity and efflux transporters, allowing the influx of immune cells, for instance, dendritic cells, neutrophils, and monocytes, as well as the secretion of proinflammatory cytokines into the CNS [197]. These cytokines will further affect astrocytes and endothelial cells by stimulating the expression of MMPs, adhesion molecules, chemokines, and cytokines, disrupting the BBB [198]. Moreover, cytokines serve as a chemoattractant for immune cells and induce signalling cascades that further upregulate their expression, resulting in neuroinflammation [199]. An inflammatory *in vitro* BBB model can be mimicked by integrating inflammatory mediators with BBB endothelial cells and including significant producers of inflammatory cytokines such as astrocytes, microglia, neurons, oligodendrocytes, monocytes, and neutrophils [200].

In a transfected human brain microvascular endothelial cells (THBMEC)-based *in vitro* BBB model, IL-1β upregulates the mRNA expression of intracellular adhesion molecule-1 (ICAM-1) more than TNF-α. IL-1β induces protein expression and the secretion of IL-1β, IL-6, IL-8, TNF-α, and MMP2, leading to TEER values decreasing by approximately 35% and increasing paracellular permeability. IL-1β also significantly increases the number of transmigrated peripheral blood mononuclear cells (PBMCs), T-lymphocytes, and monocytes by 50%. TNF-α treatment, on the other hand, did not affect the THBMEC layer [172]. Comparatively, the cytokines and chemokines of NT2 human astrocytes mediate human leukocytes (monocytes, NK cells, B cells and T cells) migration by more than 500% [201].

Shigemoto-Mogami *et al*. reported that a rat *in vitro* BBB model (RBT-24H), comprising endothelial cells, pericytes, and astrocytes, incubated for one day with LPS-activated microglia (LPS-MG) to the basolateral side, had a significantly reduced TEER value and increased paracellular transport of sodium fluorescein [202]. The expression levels of occludin and ZO-1 were significantly reduced, indicating that microglial activation could prompt BBB disruption during neuroinflammation. The concentrations of 19 cytokines/chemokines were significantly increased by LPS-MG including GRO/KC, LIX, IP-10, MIP-1α, MIP-2, MCP-1, RANTES, fractalkine, leptin, IL-1α, IL-1β, IL-4, IL-5, IL-6, IL-10, IL-12 (p70), IL-13, TNF-α, and IFN-γ in the *in vitro* model. However, different co-cultures revealed contradictory results, indicating that the effect of cytokines/chemokines is influenced by the interactions between microglia and astrocytes rather than by activated microglia alone [202]. In contrast, Banks *et al*. discovered no significant influence of microglia, astrocytes, or pericytes on the BBB disruption induced by LPS [203]. An *in vitro* model by Burkert *et al*. reported that GM-CSF, IL-1β, IL-6, IL-8, IL-13, TNFα, MCP-1, MIP-1, and IP-10 were secreted from human NT2 astrocytes following proinflammatory activation (IL-1β and TNFα stimulation), and IL-6, IL-8, GM-CSF, MIP-1α and IL-13 were produced post-activation (by phorbol 12-myristate 13-acetate (PMA)). However, other cytokines, namely IL-2, IL-4, IL-5, IL-7, IL-12, IL-21, IFNc, LTa, and Fas ligand, were not detected under any stimulation condition [201].

In a primary mouse brain microvascular endothelial cell (pMBMEC) *in vitro* BBB model, the endothelial cell surface levels of recombinant murine ICAM-1 determined the cellular pathway of CD4+ TEM-cell (encephalitogenic CD4+ TH1 effector/memory proteolipid protein (PLP) peptide aa139-153-specific T-cell line SJL.PLP7) and caused extravasation across the *in vitro* inflamed barrier [204]. It was revealed that the CD4+ TEM cells primarily crossed the recombinant murine TNF-α-stimulated VE-CadGFP and IL-1β (0.05 ng/mL)-stimulated VE-CadGFP of pMBMEC monolayer paracellularly, whereas 52% migrated trans across the IL-1β (20 ng/mL)-stimulated pMBMEC monolayer. Therefore, this study demonstrated that endothelial ICAM-1 level on the cell surface influenced the pathway of T-cell extravasation through the BBB rather than the BBB integrity or an inflammatory stimulant [205].

In a human umbilical vein endothelial cell astrocyte-conditioned medium (HUVEC-ACM) *in vitro* BBB model, the ability of human neutrophils and monocytes to transmigrate (characterised as rate and extent of transmigration) across the endothelium was unaffected by the tightness of the endothelial BBB under non-activated and TNF-activated conditions [9]. Similarly, this was observed in transmigration across human brain TY10 and hCMEC/D3 cell lines. Disrupting the function of PECAM-1 and CD99 (*via* blocking mAbs) significantly reduced diapedesis to 20% and 15%, respectively. It was established that monocytes are predominantly paracellular (> 98%) rather than transcellular, transmigrating near the TJs. Transmigration showed no significant increase in TEER and FITC-dextran permeability [9].

Winger *et al*. also demonstrated that diapedesis followed a similar pathway in high-TEER BBB endothelium and conventional endothelium, with gap formation in VE-cadherin distribution. Claudin-5 and VE-cadherin gaps were modified after the diapedesis completion of monocytes. These findings established that diapedesis is a tightly controlled process, providing a new understanding of endothelial-leukocyte interaction at the barrier and implying that TJs are more dynamic than previously recognised [9].

## 4 *IN VITRO* BLOOD-BRAIN BARRIER MODEL IN STUDIES OF NEUROINFECTIOUS DISEASES

Experimental cell culture models are required to understand the pathophysiology of the BBB and to investigate the trafficking of parasites, viruses, fungi, bacteria, immune cells, or drugs into the CNS, which will eventually result in the establishment of protective and therapeutic methods to inhibit microorganism access or penetration across the BBB. The migration of trans-endothelial leukocytes across an impaired brain microvasculature is a noted feature of many neuroinflammatory diseases. It is ambiguous whether the immune cells cross the BBB *via* TJs, a vacuole, a large pore, or other mechanisms [204]. The *in vitro* BBB model has been applied to study the pathophysiology of many inflammatory and infectious neurological diseases, including meningitis, multiple sclerosis, and neurocysticercosis, but with varying results. The impact of the selected diseases on BBB parameters is illustrated in Fig. (**1**). The *in vitro* BBB model applications in neuroinfectious diseases studies, which are discussed in this section, are summarized in Table **6**.

### 4.1 Bacterial Infection

#### 4.1.1 Escherichia coli Meningitis

Disruption of the BBB is widely regarded as a hallmark of the pathogenesis of bacterial meningitis but with an unknown mechanism [9]. *Escherichia coli* (*E. coli*) meningitis was hypothesised to occur because of a bacterial invasion of the barrier, which leads to the development of CNS infection [206].

In a human hBMECs *in vitro* BBB model, the meningitic *E. coli* PCN033 strain (at the multiplicity of infection (MOI)=10, ~ 10^8^ colony-forming units (CFUs)) significantly upregulated the transcription of platelet-derived growth factor-B (PDGF-B) and ICAM-1, which reduces any accompanying infection [207]. PDGF-B reduced the TEER values of the hBMEC monolayer and downregulated the mRNA levels of TJP expression, including ZO-1, occluding, and claudin-5 [207]. Apart from PDGF-B, the meningitic *E. coli* PCN033 strain also showed evidence of enhanced Snail-1 and VEGFA upregulation *via* TLR2-MAPK-ERK1/2 signalling pathway in an *in vitro* hBMEC monolayer and mice models [105]. Similar to PDGF-B, the expression of Snail-1 and VEGFA also downregulates the expression of TJP, leading to an increase in the permeability of BBB. Meningitic *E. coli* infection resulted in a significant secretion of GRO-α, MIP-2, MCP-1, IL-1β, IL-6, and TNF-α, which also enhanced the upregulation of Snail-1 and VEGFA, further accelerating the BBB damage. These findings demonstrated the involvement of ICAM-1 and PDGF-B in bacteria-induced BBB disruption and neuroinflammation, indicating that they could contribute to the prevention and treatment of bacterial meningitis [207], whereas Snail-1 and VEGFA serve as critical targets for meningitic *E. coli*, which eventually induced CNS destruction [105].

Another meningitic *E. coli* strain (RS218 (O18:K1:H7)) with specific virulence factors (NlpI, OmpA and FimH) was reported to upregulate the expression and release of heparin-binding epidermal growth factor-like ligand (HB-EGF) in an *in vitro* model. The HB-EGF then lead to epidermal growth factor receptor (EGFR) transactivation, which depends on the sphingosine kinases 2 (SphK2)-sphingosine 1-phosphate (S1P) (SphK2-S1P-S1P2) signalling cascade. However, the underlying mechanisms are still ambiguous [208].

#### 4.1.2 Pneumococcal meningitis

Two clinical strains of *Streptococcus pneumoniae* (*S. pneumonia*) serotype 19F (RSP1 isolated from gastric infection and RSP2 from an ear infection) demonstrated increased adherence and invasion (MOI=1) abilities in an *in vitro* bEnd.3 model compared to the control strain. Researchers determined that the *S. pneumoniae* strains isolated from the ear and gastric infection sites can cause severe secondary infections, such as bacterial meningitis [184]. No difference in VE-cadherin staining was observed in HBMEC after incubation with *S. pneumoniae* TIGR4 (10^6^ CFU) for an hour. Homogenous expression of VE-cadherin was observed at intercellular junctions in the juxtaposition of attached pneumococci, implying that the interaction between *S. pneumoniae* and the BBB does not result in a significant disruption of endothelial integrity [209]. The 37/67-kDa (kilodalton) laminin receptor (LR) was recognised as a common receptor for *Haemophilus influenza* (*H. Influenza*), *S. pneumoniae* and *Neisseria meningitides* on the surface of the mouse and human BMECs in both *in vivo* and *in vitro* studies. It is likely that inflammation during the bacteremia preceding the onset of meningitis plays a crucial role in upregulating LR prior to the LR-bacteria interaction. Furthermore, mutagenesis studies revealed that the correlated bacterial LR-binding adhesins were OmpP2 of *H. influenzae*, pneumococcal CbpA, meningococcal PorA (Porin protein), and PilQ (Pilin accessory protein). This interaction could facilitate infection transmission throughout the body since LR is commonly expressed in cell types in the human organs [210].

#### 4.1.3 Streptococcal meningitis (Neonatal Meningitis)

It was discovered that Group B *Streptococcus* (GBS) COH1 binds to both the cell surface and immobilised fibronectin, a critical ECM component of the barrier and a ligand for the β1-integrin family [211], forming a streptococcal fibronectin-binding protein A (SfbA). The SfbA expression in the non-invasive strain of *Lactococcus lactis* was adequate to enhance the binding of fibronectin and the invasion of hBMEC. Furthermore, inhibiting fibronectin binding to integrins with an anti-fibronectin antibody caused a significant reduction of the invasion of the wild-type strain but not the SfbA-deficient mutant strain, indicating that the interaction of SfbA-fibronectin-integrin is required for cellular invasion of GBS (MOI=1 and 10). Another study showed that wild-type GBS adhered to, invaded, and stimulated induced pluripotent stem cells (iPSC)-derived BMECs and exacerbated the disruption of the tight junction proteins, whilst mutant GBS, with impaired interaction, was attenuated in the iPSC-derived BMEC model [212]. These findings indicate that GBS-SfbA has a crucial contribution to the interaction between BBB endothelium and GBS and streptococcal meningitis pathogenesis [213].

### 4.2 Viral Infection

Viruses are silent reservoirs within the body that have the potential for tissue destruction. A CNS infection presents a particular challenge due to the strict control of the BBB. Neuroinvasive viruses that can cross the BBB include tick-borne encephalitis virus (TBEV), Japanese encephalitis virus (JEV), and West Nile virus (WNV). Although the molecular determinants underlying the neuroinvasive phenotype remain unknown, the *Visna virus* and HIV have shown evidence of crossing the CNS intracellularly by invading infected cells [214].

#### 4.2.1 Coronavirus Disease (COVID-19)-associated Neurological Disease

The severe acute respiratory syndrome coronavirus 2 (SARS-CoV-2) infects humans *via* the widely expressed angiotensin-converting enzyme 2 (ACE2) receptor [215, 216], which is found on a diversity of cells, for instance, alveolar type-2 cells, glial cells, brain endothelial cells, and neurons [217-221]. Human BMVECs have also been shown to express the ACE2 and NRP1 receptors [218, 220], making them possible targets for SARS-CoV-2 infection [220]. SARS-CoV-2 could infect the CNS due to the presence of virus entry receptors (ACE2, NRP1, and BSG) and viral modification (TMPRSS2, CTSL, and others) in the human brain [221].

An early *in vitro* study of the coronavirus JHM OMP1 strain revealed that it was capable of infecting cultivated BMVECs from rhesus macaques and humans [222], implying that the virus could cross the BBB. A more recent study discovered that the human umbilical vein endothelial cells (HUVEC) had a high level of ACE2 expression. The S1 subunit of the SARS-CoV-2 spike protein (11.1%) was present in the HUVEC cells and endocytosed HUVEC cells, which co-localised with caspase 3 (the isolated spike and isolated caspase 3). Increased HUVEC adhesion and degeneration and a significant increase of spike subunit and caspase 3 cells (in percentage) were observed in increasing the S1 spike protein concentration (70 ng/ml to 350 ng/ml) [223].

Immunofluorescence labelling revealed that after a SARS-CoV-2 spike protein administration, basal ACE2 receptor expression was dramatically elevated in BMVEC. Researchers discovered that the primary human BMVEC express ACE2 receptors as exposure to SARS-CoV-2 spike protein increases [224]. Using the ohmmeter Millicell ERS system, a significant drop in TEER was seen in a 2D BBB *in vitro* co-culture Transwell model treated with SARS-CoV-2 spike protein (0.5 g/ml) with a 29% decrease in TEER and or heat-inactivated SARS-COV-2 (5 μl/ml medium) with a 30% decrease in TEER value. These findings imply that SARS-CoV-2 exposure changes BBB integrity, allowing neuroinvasion [224]. Furthermore, gene expression levels analysed by real-time quantitative PCR reported TJPs JAM-2 (45%), ZO-1 (52%), ZO-2 (92%), and claudin-5 (97%) to be significantly lower in recombinant SARS-CoV-2 spike protein-treated BMVEC, compared to JAM-2 (85%), ZO-1 (69%), ZO-2 (86%), and claudin-5 (95%), heat-inactivated SARS-CoV-2 spike protein-treated. This showed that SARS-CoV-2 could alter gene expression of TJP and or post-translational modifications, hence impairing the integrity and function of the BBB. TNF-, IL-6, IL-10, IL-23, and IL-33 production were also found to be significantly increased, indicating a considerable rise in pro-inflammatory cytokines that, through the ACE2 receptor, would trigger endothelial inflammation and compromise the BBB’s integrity in BMVEC cells, thereby allowing SARS-CoV-2 neuroinvasion [224]. Therefore, findings from this study revealed that SARS-CoV-2 might penetrate the BBB through non-specific endocytosis or spike protein-ACE2 interactions, resulting in neuroinflammatory reactions and neuropathology [224].

ACE2 was expressed in primary hBMVECs and hCMEC/D3 cells in another study. However, in these cells, the spike protein subunits (subunit S1 or S2) had no effect on ACE2 expression. Only chronic exposure to the SARS-CoV-2 spike protein (> 72 h) resulted in a significantly higher rate of cell death [225]. These results corroborated recent clinical evidence indicating endothelial cell loss in severe infections of COVID-19 [225].

The spike protein subunits caused a dose-dependent decrease in the electrical resistance of the hBMVECs' barrier, which peaked at 12-14 hours (using ECIS). Both the SARS-CoV-2 subunits S1 and S2 induced comparable effects in addition to a temporary reduction of electrical resistance that was regained entirely after 24 hours, suggesting that structural reconfiguration occurred rather than an evident loss of tight junctional complex. The rate of passive paracellular penetration of small tracer molecules (4 kDa FITC-dextran) significantly increased with each additional SARS-CoV-2 spike protein subunit, providing another signal of barrier disruption. The SARS-CoV-2 spike protein is believed to have the ability to generate a low-grade chronic disruption of BBB that is time and concentration-dependent. Increased barrier permeability and decreased ZO-1 localisation to cell-cell junctions in a 3D human BBB model confirm the results of the 2D BBB model, suggesting that the subunit S1 of SARS-CoV-2 is responsible for BBB failure [226].

The fact that S1 and S2 subunits and receptor-binding domain (RBD) have similar impacts on barrier function is intriguing because it indicates that the SARS-CoV-2 - brain endothelial cells interaction is complex and not dependent on ACE2 receptor alone. The SARS-CoV-2 -BBB interaction is most likely multifocal, including reversible activation of several receptors or signalling pathways [226]. Moreover, the increased ECM enzymes (MMP3), cell adhesion molecules (ICAM-1 and VCAM-1) and endothelial pro-inflammatory response (CCL5, CXCL10) in the human endothelial cells further suggest that SARS-CoV-2 is possibly a neuroinvasive virus since it activates the mechanism that facilitates the migration of infected immune cells into the brain parenchyma as “Trojan horses” [226].

SARS-CoV-2 could induce neuroinflammatory responses without any virus entering the target cell cytoplasm, as the virus degradation in the lysosome or endosome can activate danger-associated molecular patterns (DAMPs) and or pathogen-associated molecular patterns (PAMPs) to induce immune response [227]. Coronaviruses may also reach the CNS through a compromised BBB caused by intracellular virus transmigration of macrophages, inflammatory mediators, brain endothelial damage or endothelitis, or direct infection of brain endothelial cells [228-231].

#### 4.2.2 Human Immunodeficiency Virus (HIV-1)-associated Neurocognitive Disorder

Despite the development of potent antiretroviral therapy, human immunodeficiency virus-associated neurocognitive disorders (HAND) continue to be among the mainly frequent disorders presented in HIV-infected individuals. HAND refers to a severe form of CNS association, including asymptomatic neurocognitive impairment (ANI), minor neurocognitive disorder (MND), and more severe HIV-associated dementia (HAD) [232]. HAND is frequently linked to HIV morbidity, including the presence of a ‘hidden epidemic’ in the CNS [233].

A study from McFarren *et al*. showed that 213Bi-2556 mAb (Human IgG1 mAb 2556 to gp41) could cross a human BMVEC-astrocytes *in vitro* BBB model *via* transcytosis and mainly kill transmigrated HIV-infected PBMCs and monocytes (CD14+CD16+ cells) without apparent disruption of the BBB [234]. In a human BMVEC-brain pericytes co-culture model with monocyte adhesion/migration, it was discovered that there was reduced expression of Cx43, α-smooth muscle actin (a-SMA), α1-integrin, and platelet-derived growth factor-B receptor (PDGF-Rβ) (pericytes markers) [235]. This was followed by the increased number of filopodia after exposure to TNF-α or IL-1β, indicating the development of a migratory phenotype that has less coverage of the barrier. Pericytes stimulation by pro-inflammatory cytokines (IL-1β and TNF-α) associated with HIV neuropathogenesis and HIV-1 led to an increase in inflammatory factor secretion, adhesion molecules upregulation, and a migratory phenotype, altogether possibly promoting to the disruption of BBB [235].

Furthermore, assessed alteration in mRNA expression of basement membrane components, fibronectin, and nidogen showed a downregulation (60%) in stimulated pericytes and a significant reduction (25%) in metalloproteinases-3 (TIMP3), a tissue inhibitor. These results imply possible pericyte dysfunction, including the expression of cellular markers and receptors and the production of BBB-supporting factors, which may contribute to BBB disruption. A significant increase (7-8% high) in the TEER of BMVEC-pericyte cultures was observed post-incubation with primary human monocytes. Monocyte migration to the abluminal of BMVEC was increased 6-7-fold when BMVEC and pericytes were co-cultured following CCL2 addition, a cytokine upregulated in HIV-1 infection of the CNS [236], showing increased BBB “tightness” in a co-culture with pericytes. Pericytes stimulation with TNF-α increased upregulation of ICAM-1 (7-10-fold) and vascular cell adhesion molecule-1 (VCAM-1) (4-7-fold) expression. These findings indicated that pericytes might play a role in monocyte penetration across the barrier and retention in perivascular spaces [235].

#### 4.2.3 Viral Encephalitis

Viral encephalitis is the inflammation and swelling of the brain, which can be caused by enteroviruses, adenovirus, Epstein-Barr virus, Japanese encephalitis virus, and many more [237]. In HBEC-5i cells-primary astrocytes *in vitro* BBB model, infection by Semliki Forest virus (SFV6) and SFV6-162K strains (MOI=0.1) caused a significant decrease in TEER. The capability of SFV to cross the *in vitro* BBB is related to its ability to replicate in the brain, but not the capability to produce a high-titre viremia [214].

It was also reported that glutamic acid elevates the viral load in mouse blood plasma, neuroinvasion and virulence. Lysine promotes the capability of the virus to traverse the BBB and replicate in the brain. Viruses with the dominant dependence on binding to glycosaminoglycans (GAGs) replicate to higher titres in the brain and can traverse the *in vitro* BBB [214].

In another HBEC-astrocytes *in vitro* BBB model, Japanese encephalitis virus (JEV) strain CNS138-11 could infect (MOI=1) both HBECs and astrocytes and elevate BBB permeability (approximately mean TEER 100 to 50) with limited active viral production in endothelial cells before traversing the barrier and infecting astrocytes. This enhanced permeability has been interpreted as a further viral entry pathway into the CNS. HBECs act as the initial barrier to JEV transmigration. JEV may infect and replicate HBECs prior to infiltrating the brain and stimulating a robust pro-inflammatory response in HBECs and astrocytes [13].

The release of CCL5, CXCL10, and IL-6 upon JEV infection elevated with time and was associated with TEER. Numerous factors, including CXCL10, MMP7, VCAM, leptin, and IL-6, showed significant correlations with TEER. However, releasing these factors may exacerbate BBB disruption and provide an entry point for the JEV to cross into the brain [13].

Comparatively, in an HBMVE *in vitro* BBB model, WNV (strain NY99), infection (MOI=1 and 5) in HBMVE cells did not cause a gross cytopathic effect. However, elevated expressions of mRNA and protein of claudin-1, VCAM, and E-selectin were observed post-infection. The cell-free penetration of WNV through the barrier *via* the transcellular pathway, rather than the paracellular pathway, did not compromise the BBB integrity, and post-infection TEER values and FITC-dextran percentage did not differ significantly. This could be due to the possibility that WNV-infected HBMVE cells help the virus enter *via* the ‘Trojan horse’ mechanism, in which infected monocytes or macrophages travel into the CNS and transmit WNV into brain cells [238].

Another HBMVE-HBCA *in vitro* BBB model proved that WNV strain NY99 could efficiently replicate in HBMVE cells [6]. WNV infection is vulnerable to human brain cortical astrocytes (HBCA) (MOI=5), whereby WNV promoted mRNA and protein expression of MMP-1, -3, -9 in HBCA cells (MOI=5 and 3). It was strongly suggested that virus replication and virus entrance triggers MMP production. ZO-1 protein decrease was observed in HBMVE cells treated with the WNV-infected HBCA cells' supernatant and increased expression of claudin-1. In the presence of an MMP inhibitor, the integrity of the BBB model damaged by MMPs released by WNV-infected HBCA cells can be restored. BBB models incubated with the WNV-infected HBCA cells' supernatant had their TEER decreased significantly (from 650 Ω/cm^2^ to ~400 Ω/cm^2^), while their FITC-dextran transmigration increased significantly (from 2%-3% to 190%). These findings suggested that the brain astrocytes produce MMP, which causes BBB breakdown, allowing immune cells unlimited access to the brain and leading to WNV-neuropathogenesis [6].

#### 4.2.4 Dengue Virus Infection

The cerebrovascular endothelium’s involvement and the alteration of the BBB associated with neurological symptoms following dengue virus infection and dissemination in the CNS are unknown [239]. Only one study in a neonatal mouse brain endothelial cells (MBECs) and MBECs-astrocytes *in vitro* BBB model showed that they are susceptible to infection by both DENV-4 and D4MB-6 variants (MOI=1) and are characterised by perinuclear-localized viral E protein [239].

A disruption in the endothelial barrier permits paracellular transport of the dengue virus. These findings demonstrate that the early stages of infection (transcription-translation of viral proteins and viral entrance) resulted in significant alterations to the BBB functioning, allowing the transcellular or paracellular transport of free viruses from the apical to the basolateral of the filter support [239].

Both virus DENV-4 and D4MB-6 strain infections cause a significant increase in blue dextran concentration (30% in DENV-4-infected cells and 60% in D4MB-6-infected cells), concurring with the changes in TEER (34% in DENV-4-infected cells and 44% in D4MB-6-infected cells). TEER loss (18.1% in DENV-4- and 16.3% in D4MB-6-infected cells) is less severe and resulted in a slight increase in blue dextran concentration (less than 1%) in the MBECs-astrocytes co-culture model. These findings reveal that astrocytes play a protective role in brain endothelial responses during a dengue virus neuro-infection [239].

Dengue virus infection also prompts alterations in the subcellular-localized ZO-1 and Claudine-1. A DENV-4 infection has no effect on the MBEC cells with a ZO-1 pattern and in both BBB models. In contrast, the D4MB-6 virus influences the re-localization of ZO-1 from the cell border to the cytoplasm, causing an interrupted linear fluorescence pattern in the membrane. Although both viruses promote detachment of cells, the monolayer model of MBEC was reported to be extended effective in terms of cell loss and complete ZO-1 re-arrangement [239].

Furthermore, D4MB-6 infection promotes brain endothelial activation and monocytes/macrophages transmigration. D4MB-6-infected endothelial cells in the MBEC-astrocyte co-culture model permitted significantly higher migration of J774 macrophages, even when these macrophages were not infected. It is suggested that a Trojan horse mechanism is exploited late in the infection stage. This mechanism, together with viral paracellular transport, is the primary strategy for the dengue virus to gain entry into the CNS [239].

#### 4.2.5 Zika Virus Infection

Zika virus (ZIKV), a mosquito-borne flavivirus, is neurotropic in infants [240] and adults [241]. In an *in vitro* BBB model derived from human iPSCs, it was demonstrated that ZIKV isolate 2013 (*i.e*., Asian lineage) could cross the *in vitro* barrier through the iPSC-derived induced brain endothelial cell (i-BEC), induced neural progenitor cells (i-NP), and i-Ns, and thus infect neural cells [186].

The ZIKV (MOI=4) can cross the human intact BBB model (*via* measuring ZO-1, TEER ≥ 300 Ω.cm^2^) without affecting its sodium fluorescein permeability paracellular nor the i-BEC monolayer viability. AXL, a well-characterised ZIKV entry receptor, is overexpressed in i-BEC and i-NPs with ZIKV susceptibility. Thus, the primary mechanism by which ZIKV crosses the BBB has been hypothesised to involve infection of i-BEC, transcellular crossing, and release onto the monolayer’s abluminal surface [186].

#### 4.2.6 Hepatitis C Virus Infection-associated Neuropathology

Hepatitis C virus (HCV) is primarily a hepatotropic virus that could result in a range of extrahepatic conditions, including CNS disorders and progressive liver disease [242]. HBECs facilitate the entry and replication of HCV (HCV envelope glycoproteins (HCVpp) and vesicular stomatitis virus G glycoprotein (VSV-Gpp)) in an *in vitro* BBB model elucidating a possible pathway for an HCV infection of the CNS. All crucial HCV receptors are expressed on hCMEC/ D3 and HBMEC [243], facilitating the entrance of HCVpp.

Antibodies of SR-BI, CD81, claudin-1, and E2 glycoprotein dramatically inhibited infection of hCMEC/D3 cells, demonstrating that cellular CD81 and virus-associated ApoE have a role in the transmission of virus between HBECs, and a receptor-dependent entry route similar to that found in hepatocytes and cell lines derived from hepatocellular carcinoma [243, 244]. Infection of hCMEC/D3 results in increasing HCV RNA and antigen expression gradually, resulting in cytopathic effect and increased paracellular permeability marker fluorescein isothiocyanate/dextran 70 kDa, hence demonstrating that HCV has the potential to compromise the integrity of brain endothelial cells. This is further proved when HCV infection is neutralised by anti-HCV patient immunoglobulin pooled, restoring hCMEC/D3 permeability and suggesting a direct effect of infection [245].

### 4.3 Parasitic Infection

*In vitro* BBB models allow for direct research and utilization of the molecular pathways behind African trypanosome transmigration into the brain. It was demonstrated that two human pathogenic strains (*Trypanosoma brucei rhodesiense* and *Trypanosoma bruc*ei *gambiense*) and *T. b. brucei* TC221 could traverse the optimised ECV304-C6 BBB. These findings indicated that the J774 macrophage transmigration had no synergistic effect on T. b. brucei TC221 traversal. A tenfold (10^6^ to 10^7^ parasites/insert) increased trypanosomal inoculum resulted in an increase in T.b. brucei and T.b. rhodesiense transmigration but not in T.b. gambiense transmigration, showing that a particular density of trypanosomes is requisite to be reached to achieve the highest rate of transmigration. This study demonstrated that parasites traversed an inverse ECV304-C6 BBB model with comparable transmigration rates to the conventional BBB model [246].

#### 4.3.1 Cerebral malaria

Cerebral malaria (CM) is a severe infection caused by *Plasmodium falciparum*. In *in vitro* endothelial *Plasmodium berghei* ANKA (PbA) cross-presentation model, primary MBECs activated with IFNγ were destroyed by infected mouse CD8+ T lymphocytes when grown with parasite-infected red blood cells [247]. No cross-presentation was observed when PbA mature stages (merozoites) were isolated from MBECs using Transwell inserts, indicating that the cross-presented antigen source was not a soluble protein. PbA were phagocytosed and cross-presented more effectively than parasite strains without resulting in *in vitro* CM, inferring that free merozoites were probably the primary source of cross-presented antigen [247]. The mouse brain endothelial cells primarily phagocytosed digestive vacuoles and free merozoites. Immortalised hCMEC/D3 cells phagocytosed merozoites with high avidity, implying that brain endothelial cells' cross-presentation of parasite antigens may contribute to the human CM pathogenesis [247].

In a co-culture of *Plasmodium falciparum* RAOL isolate-parasitised human red blood cells (PRBCs) and hCMEC/D3 cells model, cytoadherence of PRBCs was mediated by a trypsin-sensitive antigen (60% parasitaemia and 2.5% haematocrit), as trypsin treatment significantly reduced PRBCs' capacity to attach to endothelial cells. These findings suggested that cytoadherence was mediated by a trypsin-sensitive antigen expressed by *P. falciparum* RAOL as well as hICAM-1 expressed by (TNFα)-stimulated hCMEC/D3 cells [248]. Furthermore, PRBCs (50% parasitaemia and 2.5% haematocrit) and merozoites demonstrated the ZO1 disorganization immunostaining with spaces between cells and ZO1 relocalisation in the intracellular compartment of the hCMEC/D3 co-culture. A higher dose of the parasite (8 x 10^7^ parasites/cm^2^) significantly increased the permeability of endothelial cells in this model, and PRBCs-induced permeability of endothelial cell alteration showed contact-independent and cytoadherence mechanism through the decrease of the environmental pH of the culture (acidosis) [248].

### 4.4 Neurocysticercosis

Secretory and excretory antigens of *Taenia solium* cysticerci cause angiogenesis in an *in vitro* HUVEC model by endothelial cell tube formation assay [108]. The *in vitro* HUVEC model supports the theory that angiogenesis in neurocysticercosis (NCC) is generated by the parasite rather than the host's inflammatory reactions. Upon infection, there was the presence of brain vascular changes, BBB disruption (EBA and IgG), overexpression of angiogenesis markers (VEGF-A and FGF2), and surrounding viable cysts. Vessels revealed EBA marker reduced immunoreactivity and widespread IgG staining in the tissues around the cysts. In astrocytes, VEGF-A expression was increased, reflecting the possible involvement of astrocytes in the pathogenesis of NCC [108].

A study by Lachenmaier *et al*. applied an *in vitro* rat brain endothelial and glial cells BBB model to investigate the possibility of *Toxoplasma gondii (T. gondii)* migrating intracellularly rather than extracellularly through the BBB in a ‘Trojan horse’ mechanism. Regulated genes related to cell adhesion (E-selectin, P-selectin and ICAM-1), toll-like receptor (TLR) 4, tight junctions, cytokines (IL-6) and chemokines (CCL2 (MCP-1), CCL7 and CX3CL1) suggest that the neuroinvasion of *T. gondi)* GFP+ tachyzoites RH strain (type I) and ME49 strain (type II) in bEnd. 3 cells may involve the utilization of leukocyte transendothelial migration pathways. Additionally, the infected CD45+/CD11bc+ (antigen-presenting) cells percentage significantly increased to 4.63% after migration compared to 0.6% of infected CD45+/ CD11bc− (lymphocytes) cells. Therefore, *T. gondii* was likely to be transported across the barrier through these antigen-presenting cells [249].

The CD11b+ cells were also the predominant cells migrating through the barrier upon infection with GFP+ tachyzoites (MOI=1) with upregulation of ICAM-1, enabling the leukocyte migration across endothelial barriers in the rat *in vitro* BBB model [249]. Meanwhile, compared to the RH strain (type I) tachyzoites, the ME49 strain (type II) showed significant early infectivity post-infection in bEnd.3 cells simultaneously stimulated the ICAM-1 overexpression and IL-6 and MCP-1 high secretion. Tachyzoites of the ME49 strain tended to infect more CD11b, CD11c, or both cells prior to migration, implying that Toxoplasma strains type I or type II would produce similar migratory cell patterns through the *in vitro* BBB [249].

## 5 OTHER APPLICATIONS OF *IN VITRO* BLOOD-BRAIN BARRIER MODEL

*In vitro*, BBB models have several applications for studying the BBB's structure and function in health and diseases, drug research, and toxicology, including facilitating the study of endothelial cell-leukocyte interaction and the mechanisms underlying immune cell extravasation into the brain. A detailed analysis of the adhesion molecules involved in the recruitment of specific subsets of immune cells into the target organ/tissue could lead to a way for reducing organ-specific inflammation without impairing immunosurveillance.

Such standard and validated models can be used to examine the effects of bacterial and or viral infections, as well as the migration of bacteria- and or virus-infected immune cells, on the permeability of the barrier [250-252]. The developed BBB model can be utilised to study pathogenic mechanisms, hence allowing for the investigation of a broad range of molecular or signalling pathways. Ultimately, studying and developing an optimal and valid BBB model may provide a novel insight into future prevention and a non-antibiotic therapy of pathogenic meningitis [207].

## 6 ADVANTAGES AND LIMITATIONS OF *IN VITRO* AND *IN VIVO* MODELS OF BLOOD-BRAIN BARRIER

The understanding of the BBB is based on the correlation between *in vitro* and *in vivo* models and clinical investigations on humans. *In vivo* animal models can recapitulate the pathological conditions of neuroinfectious diseases in complicated settings. *In vivo* modelling of BBB physiology captures the multicellular and hemodynamic components of the BBB [253, 254]. Understanding alterations in the BBB and simulating the transport of neurotherapeutics have relied heavily on animal models. Animal models have enabled the in-depth study of the anatomy and biology of the BBB, which is typically only attainable in humans utilising post-mortem tissue. Critical for understanding the role of the BBB at various phases of illness progression, post-mortem tissue does not permit the examination of BBB structure and function in a living individual and at early disease stages [123].

*In vitro* models of the human BBB are essential for overcoming species variations in BBB modelling and expanding the understanding of the biology of BBB (at the cellular level) in health and illness [255]. *In vitro* models of the human BBB are commonly used to boost experimental throughput and permit the examination of a wider variety of biological factors in a human-relevant system [256]. Animal and clinical research are frequently preceded and complemented by *in vitro* BBB models [257, 258].

Preclinical investigations have drawbacks, such as (a) frequently time-consuming and (b) costly, (c) multiple animals are required to generate statistically significant data, (d) ethical problems, and (e) they may lack translational relevance to the human condition [254], This is due to, even though animal models of neuroinfectious diseases express crucial pathological markers, they do not necessarily reflect other biological characteristics of diseases. There are also insufficient animal models for sporadic forms of human disease [259].

The limitations of *in vivo* models are due to (a) species variations at the genomic (10% difference between human and mouse, 3000 genes) and molecular levels (species-specific expression level of specific proteins) [260]. For example, the response of mice and humans to cytotoxicity or inflammatory stimuli at the barrier [261]. Such differences could impact disease targeting and treatment tactics. A study comparing the expression of genes in BECs of mice and humans discovered a cluster of human-specific genes but none in mice [262]. This may directly impact the pathogenesis of neuroinfectious diseases and hinder the success of animal disease modelling, (b) There are also species-specific variations in protein function, such as the substrate affinity of specific ABC transporters [128], (c) transporter and TJP expression and function differ between the BBBs of rodents and humans. Important TJPs, including ZO-1, occluding, and claudin-5, have greater mRNA expression in mouse BECs than in human BECs [263]. Moreover, a comparison of the protein transporter expression levels in brain microvessels revealed that the expression of specific transporters, such as ABC (P-gp and MRP4) as well as solute carrier transporters (organic anion transporter 3, L-type amino acid transporter, and monocarboxylate transporter 1), was significantly higher in rats than in humans [264]. Positron emission tomography (PET) showed differences in P-gp-dependent drug uptake between rats and humans, with greater P-gp substrate concentrations in human brains than in rat brains [126], (d) O’Brown *et al.* (2018) describe significant changes in the morphology and gene expression of neocortical cells between the mouse and human. Moreover, the human neocortex is considerably larger and more intricate than the mouse neocortex, which exacerbates medication administration in humans [265]. In addition, the number of astrocytes in the human neocortex is proportionally higher than in the mouse cortex [266], which may influence BBB development and subsequent drug transport. Other NVU cell components have also been shown to exhibit interspecies variations. Rat astrocytes are smaller and have a different process complexity than their human counterparts [67].

*In vitro* BBB models have “design/manufacturing” limitations, practical limitations, and measurement limitations, such as (a) limited mimicry of BBB and microenvironmental characteristics, *e.g*., cell-cell/cell-matrix interactions that modify transport exchange mechanisms, (b) inaccurate brain capillary models as a result of inadequate junctional protein and membrane transporter expression, as well as (c) the need to modify culture conditions for each model, ECs might distribute inhomogeneously on the membrane, resulting in poor barriers, (d) efflux functionality and barrier tightness must be improved, (e) current models have larger channel diameters (100-800 m) than *in vivo* BBB vasculature (capillaries 7-10 m) [165, 267-269].

## CONCLUSION

Several high-quality *in vitro* BBB models have been developed for the *in vitro* investigation of neuropathological diseases. This review article has been prepared and constructed to discuss the numerous parameters involved in optimising, validating, and maintaining an *in vitro* BBB, which can serve as a platform and reliable standard system for developing a functional *in vitro* BBB model in the future. It is well established that the disruption of the BBB accelerates the course of CNS diseases. Therefore, *in vitro* BBB models are a vital tool to study bacterial, viral, and parasitic invasions of the CNS. We summarised the pathogenic mechanisms implicated in BBB penetration by pathogens that commonly cause neurotrophic disorders in humans. A significant challenge is to apply all of these parameters to simplify our understanding of disease pathogenesis, provide valuable intuitions for improving BBB-neurodisease modelling, modify physiological responses, and accelerate the development of novel clinical approaches and drugs to alleviate the burden of neuroinfectious diseases. In order to achieve clinical and therapeutic outcomes in the near future, it is imperative to continue theorising on neuroinfectious diseases and conducting research using improved BBB models that closely mimic the structural and functional human BBB.

## Figures and Tables

**Fig. (1):**
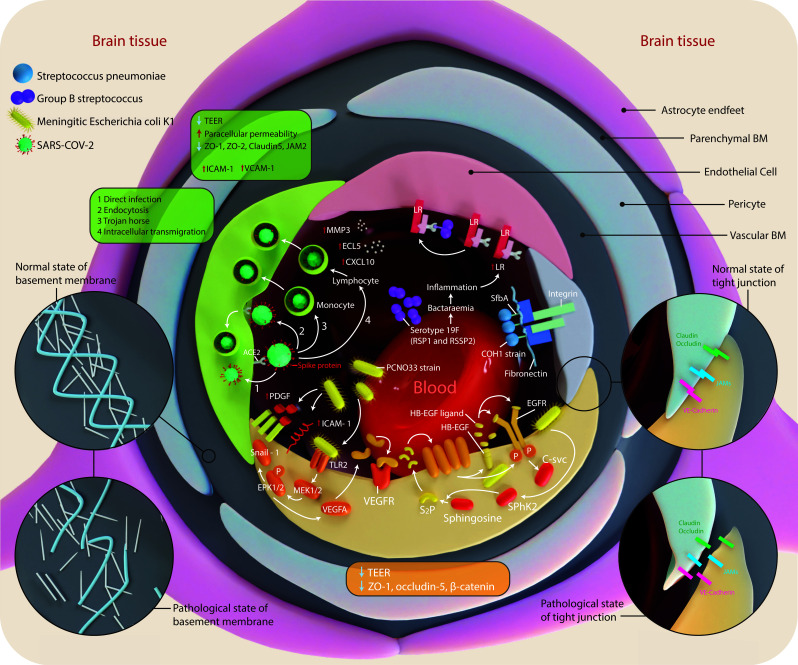
The impact of the selected diseases on BBB parameters. This figure illustrates the effect of *Streptococcus pneumoniae*, group B streptococcus, Meningitic *Escherichia coli* Ki, and SARS-CoV-2 on the BBB (attachment, tight junction and basement membrane disruption) as is mentioned later.

**Table 1:** Cellular components of the blood-brain barrier.

**Cells**	**Characteristics**	**Functions**	**Location/Position**	**Percent of Coverage**	**Key Markers**	**Secreting ** **Factors**
Endothelial cells	• Scarce pinocytotic activity [47]. • Lack of fenestrations (openings) [47]. • Unique expression patterns of trans-membrane transport systems [48]. • Have continuous intercellular TJs. • Have low expression of leukocyte adhesion molecules [49]. • Have a dynamic interaction with NVU cells. Contributing to their unique characteristics by displaying both endothelial and epithelial features [11, 16, 50].	• Physical barrier (gate function) prevents paracellular diffusion of polar molecules [16]. • Transport barrier includes several active efflux systems with affinity for lipophilic substances [16]. • Metabolic/enzymatic barrier catalyse the oxidation/metabolism of organic substrates, including xenobiotic substances such as drugs and other potentially toxic chemicals [16].	• A monolayer is lining the brain microvessels [51].	• 100%	• Adherens junction proteins: a. VE-cadherin b. PECAM • Interendothelial tight junction proteins: a. ZO-1 b. Claudins c. Occludins d. Junctional adhesion molecules (JAMs)	• PDGFβ
Pericytes	• Heterogeneous and dynamic cell population, whose expression of surface markers differs corresponding to cell differentiation and tissue distribution [52]. • Contractility [53]. • Multipotential stem cells in the adult brain [53].	• Stabilize nascent vessels and promote vascular maturation [54, 55], contributing to vessel stability, regulating capillary diameter and blood flow and controlling BBB integrity and function [28]. • May coordinate NVU assembly *in vivo* [56-58]. • The inhibition of transcy-tosis *in vivo* [56-58]. • Physical connections between pericytes and ECs have been reported transmitting mechanical forces through adhesion plaques [59] and transporting signalling molecules directly through gap junctions [28]). • Induce the polarization of astrocyte end-feet, and facilitate the attachment of end-feet to CNS vasculature [56].	• Ensheathing the abluminal surfaces of cerebral vessel walls, including capillaries, precapillary arterioles and postcapillary venules [60]. • Wrapping around BMECs on the “brain side” [61, 62].	• CNS pericytes to ECs ratio 1:3-1:1 [63]. • Contributing 22-32% of the cerebral vasculature [27].	• PDGFR-β • Α-SMA, NG2 [64].	• Angiopoietin-1 (Ang-1), transforming growth factor-b1 (TGF-b1) [28], production of basement membrane (BM) components [65].
Astrocytes	• Star-shaped glial cells. • Electrically non-excitable cells. • Heavy expression of the glial fibrillary acidic protein (GFAP) increases with age [66]. • Varicose projection astrocytes [67]. • Exhibiting a high expression of proteins involved in Ca^2+^ signalling and propagating Ca^2+^ waves [67, 68].	• Mediating signalling between neurons and BMECs [69]. • Providing nutrition for neurons, regulating extracellular potassium balance, performing neurotransmitter clearance and recycling, controlling immune reactions and regulating the BBB [70].	• Intermediary position. • Astrocyte processes are terminated in end-feets that are completely ensheathing microvessels and capillaries in the brain [69].	• 99% of the surface of brain microvessels [18]. • Glia-to-neuron ratio is a one-to-one ratio for the whole human adult brain. The human cerebral cortex has a ratio of 1.4 [71, 72]. • Approximately 20~40% of the total number of brain cells [73].	• Gap junction: Connexin30 and connexin43 [74]. ZO-1 [74]. ZONAB (ZO-1-associated nucleic acid-binding protein) [74]. GFAP and vimentin [75, 76].	• IL-6 [77]. • Glial cell line-derived neurotrophic factor [32]. • Fibroblast growth factor 2 [78]. • Transforming growth factor β1 [79].
Neurons	• Electrically excitable cells. • Mature neurons do not divide. • Neurons release neurotransmitters into synapses, or the connections between cells, to communicate with other neurons.	• The role of neurons in regulating BBB function remains poorly understood.	• Located on average, 10-20 μm from the nearest capillary [35].	• NA	• NeuN [80]. • Beta-III tubulin [81]. • Neurofilament high [81].	• Sonic Hedgehog (SHH) [34]. • Platelet-activating factor (PAF) [82].
Microglia	• Regional heterogeneity of microglial gene signatures [83]. • Complex and dynamic microglia phenotypes. • Dichotomous paradigm; represents a large spectrum of activation states and is often related to inflammatory reactions and morphological changes [84]. • Exhibiting region-specific responses [85]. • The only myeloid cells reside in the healthy CNS parenchyma [86]. • Microglia’s unique ontogeny, resident microglial cells in the healthy adult brain persist during adulthood *via* constant self-renewal without turnover from circulating blood progenitors [87, 88]. • The fastest-moving structures in the healthy adult brain, monitoring the entire brain parenchyma in less than four hours [78, 79].	• Vessel-associated microglia initially maintain BBB integrity *via* expression of the tight-junction protein claudin-5 and make physical contact with endothelial cells. During inflammation, microglia phagocytose astrocytic end-feet and impair BBB function [45]. • Microglia adjacent to the BBB are in constant bi-directional communication with endothelial cells, facilitating microglial cells to do their surveying functions on the BBB integrity and the influx of blood-derived molecules into the brain [89, 90].	• Locating at the proximal region surrounding the cerebrovasculature, allowing a close endothelium-microglia interaction [91].	• 12-16% of the total brain population [92].	• All microglia: • Iba-1 [93]. • Activated microglia: major histocompatibility complex (MHC) class I and II molecules [94, 95].	• Activated microglia: TNFα, IL-1β [96, 97] nerve growth factor (NGF), neurotrophin (NT)-4/5, transforming growth factor (TGF)-β1, glial-derived neurotrophic factor (GDNF), fibroblast growth factor (FGF), and IL-3 [98].

**Abbreviations:** NA, not available; VE-, vascular endothelial; PECAM, platelet endothelial cell adhesion molecule; ZO-1, zonula occludens-1; PDGFβ, Platelet-derived growth factor subunit B; ECs, endothelial cells; BMECs, brain microvascular endothelial cells; PDGFR-β, Platelet-derived growth factor receptor-β; Α-SMA, alpha-smoot muscle actin; NG2, neuron-glial antigen 2; Iba-1, ionized calcium-binding adaptor protein-1; IL: interleukin, TNFα, tumour necrosis factor-alpha.

**Table 2:** The advantages and disadvantages of the current models of BBB.

**BBB Models**	**Advantages**	**Disadvantages**	**References**
Endothelial monoculture	• Microscopic observation • Molecular cell analysis	• Absence of cell-cell interactions in the BBB • Endothelial phenotypes are limited • Insufficient BBB characteristics • Absent blood or media flow • Absent shear stress • Formation of monolayer	[122-127]
Transwell system	• BBB cells interaction • Enhancement of BBB's physical properties • Endothelial cell phenotypes enhancement • Formation of a stricter and more selective vascular bed • Suitable for leukocyte transmigration	• Enhancement of BBB’s physical properties • Absent of cell-cell interaction in the BBB • Absent blood or media flow • Absent shear stress	[122-127]
3D ECM-based BBB	• Utilizing self-polymerizing ECM protein scaffolds • BBB cellular components are capable of developing close connections	• It is not widely used in BBB research • The difficulty of constructing a matrix architecture resembling *in vivo* • These systems are limited to fundamental research at present	[124]
Organoid BBB	• Each cell type is in close interaction with every other cell type within the organoid • Increased expression of tight junctions, adherens junctions, and efflux pump • Ease of culture • Simple • Fewer reagents are required • Small size • Cost-effectiveness • Reproducibility	• Inter-samples variability • High processing time • The absence of essential types of cells, including glia, microglia, oligodendrocytes thus limiting its utilities for specific disease models	[87, 88, 98]
DIV-BBB	• Distribution of BBB cells topographically • Blood or media flow • Capillary-like shear stress • Enhancement of BBB’s physical properties • Enhancement BBB phenotypes	• Large vascular bed • Undistinguishable cell-cell interaction • Restricted leukocyte migration	[122-127]
Microfluid model	• Microvasculature-like microchannels • Enhancement of BBB’s physical properties • Blood or media flow • Real-time evaluation of BBB properties • Similar to physiological shear stress	• Limitation in cell-cell contact production • Fixed ECM • Limited real-time evaluation of BBB properties	[122-127]
BBB-on-a-chip	• Microvasculature like microchannels • Enhancement of BBB’s physical properties • Blood or media flow • Similar to Physiological shear stress • Real-time evaluation of BBB properties	• Reproducibility of cell phenotype and capillary size is restricted.	[122-127]

**Abbreviations:** 3D, thee-dimensional; ECM, extrasellar matrix; DIV-BBB, dynamic *in vitro* BBB.

**Table 3:** Structural, functional, and molecular characteristics of the ideal *in vitro* human BBB model.

**Structural Characteristics**
• Expression of tight endothelial junctions. • Expression of drug-metabolizing enzymes (*e.g*., P450s). • Exposure to laminal shear stress (apical membrane), glia (basal membrane), and other permissive factors induces growth inhibition and endothelial cell differentiation.
**Functional Characteristics**
• Insignificant paracellular diffusion. • Selective and asymmetric permeability to physiologically essential ions. • Functional expression of efflux pumps and selective transport systems (*e.g*., P-gp, amino acid, hexose). • Sensitivity to permeation modulators (*e.g*., hyperosmolar mannitol) and other endogenous and exogenous stimuli can alter BBB function and integrity. • Capability to imitate the effect of diverse physiological and pathological stimuli (*e.g*., hypertension, inflammation) that affect the *in vivo* BBB.
**Molecular Characteristics**
• Molecular expression of interactions between BBB cells. • Molecular expression of the junctional complex (adherens and tight). • Molecular expression of ATP-binding Cassette (ABC) and SLC Transporters of the BBB.

**Abbreviations:** P450, Cytochromes P450; SLC, solute-like carrier. Modified from [132].

**Table 4:** Optimization parameters of *in vitro* blood-brain barrier model.

**Parameters**	**Purpose**	**Property**	**Assay/Techniques**	**References**
Cell confluence, non-overlapping, uniform monolayer	• Effect on TEER. • An even and flattened morphology predominantly organised in a single layer.	Integrity	Microscopic observation, immunostaining & TEER measurement	[155, 160-161]
Cell seeding density	• Forming a functional BBB. • Cell growth and TJ development between adjacent cells. • Low or high cell seeding density caused decreased maximal TEER values.	Tightness & integrity	TEER measurement	[160, 162]
Cell viability	• Maintain the viability of primary human cells. • Maintain the viability of BBB. • Assess the impact of different factors on the cellular viability of BBB.	Tightness & integrity	Trypan blue exclusion method, MTT cellular viability assay	[122, 162, 163]
Nature of cell type	• Effect on TEER. • Confirming that the cells used are of human origin.	Mimic human BBB	DNA fingerprinting as analysed by both ICC and FC	[147, 162, 164]
Co-culturing with cellular components of the NVU	• Mimic *in vivo* BBB. • More stable. • Physiologically relevant.	Barrier function and integrity	*In vitro* BBB modelling	[162, 165]
Cell organization	• Closer representation of *in vivo* BBB. • Would be in direct contact, allowing them to exchange vital growth factors required for cellular growth and development.	Barrier function and integrity	TEER measurement	[165]
Timeline establishment of model	• Effect on TEER. • BBB physiology.	Barrier function and integrity	*In vitro* BBB modelling	[165]
Passage number of cells	• Effect on BBB phenotypes. • Dedifferentiation process.	Barrier function and integrity	Microscopic observation, immunostaining & TEER measurement	[166, 166-168]
Cultivation period	• Effect on TEER • Extended to allow the proper formation of interendothelial tight and adherens junctions.	Tightness	Immunostaining TEER measurement	[155]
**Culture Conditions**				
**Co-culture Conditions**				
Co-culture media	• Expression of endothelial markers. • Tight junction proteins. • Endocytosis machinery and P-gp efflux transporter. • High TEER. • Increased cell density.	Barrier function, tightness, integrity and improved the quality	TEER & permeability measurements, immunostaining	[169]
Cell ratio	• Increased TEER. • Improved tight junction continuity.	Performance of co-culture and multicultural models	TEER & permeability measurements, immunostaining	[131]
Cell-media volume ratios	Avoid dilution of co-culture signals and metabolites.	Performance of co-culture and multicultural models	TEER & permeability measurements	[170]
Culture surface/volume ratios	Obtaining good cell growth and differentiation.	Performance of cell culture	Cell proliferation assay and cell cytotoxicity	[170]
**Growth Medium Compositions**				
Serum (human)	• Mimic *in vivo*. • No increase in TEER could be observed. • The permeability of sucrose through hCMEC/D3 monolayers could significantly be reduced by HS supplementation.	Integrity	Permeability measurement	[160, 171]
Concentration	Low TEER values might also arise from a high concentration of serum and growth factors in the growth medium.	Integrity	TEER measurement	[155, 160]
Supplement	Medium supplemented with L-glutamine, and the reduction of the serum concentration decelerates cell growth and promotes differentiation.	Physical barrier	TEER & permeability measurements	[155]
Cell culture support materials	Allow cell differentiation, interaction and response.	Integrity	Microscopic observation, immunostaining, attachment and spreading assay & TEER measurement	[132]
**Extracellular Matrix (ECM)**				
Cell-derived matrices	Maintain some of the original gaps that accommodate the cells.	Tightness	Permeability measurement	[172, 173]
**Transwell Apparatus**				
1. Membrane materials	Impact on the adherence of cells and barrier tightness.	Barrier tightness	TEER & permeability measurements	[174, 175]
2. Pore size	Impact on the adherence and migration of cells and barrier tightness.	Barrier tightness	TEER & permeability measurements	[165, 174, 175]
3. Coating materials	Impact on cell growth and TJ development between adjacent cells.	Tightness & integrity	TEER & permeability measurements	[160]
4. Coating procedures	Impact on the adherence of cells and barrier tightness	Barrier tightness	TEER & permeability measurements	[160]
5. Well format	The selection of the well format might affect TEER values.	Barrier strength	TEER measurement	[160, 165]
6. Surface area	More surface area, more growth area.	Barrier strength	TEER measurement	[155]
**Assay/Technique**				
TEER	Real-time monitoring	Integrity	EVOM: Manual cell monitoring CellZscope system: automated cell monitoring ECIS: automated cell monitoring xCelligence: automated cell monitoring	[162]
TEER electrodes: Size, gap, orientation	Minimizing background resistance and error.	Integrity	TEER measurement	[170]
**Mechanical Factors**				
Shear stress	• Increase cell longevity. • Influence cell phenotype. • Regulate BBB transport. • Preventing de-differentiation.	Barrier function	Endothelial morphology, proliferation, apoptosis, and motility	[176-179]
Flow rates	Polarized BBB endothelium phenotype.	Barrier function	Endothelial morphology, proliferation, apoptosis, and motility	[132]

**Abbreviations:** TEER, transendothelial electrical resistance; TJ, tight junction; P-gp, P-glycoprotein; ICC, immunocytochemistry; FC, flow cytometry.

**Table 5:** Validation parameters of *in vitro* blood-brain barrier model and advantages and limitations of the methods.

**Parameters**	**Principle**	**Purpose**	**Advantages**	**Limitations**	**References**
TEER measurement	Chopstick electrodes or built-in electrodes	Tightness of BBB	• Quantitative technique • To quantify the electrical resistance • Non-invasive • Simple, rapid and reliable • TEER values are a strong indicator of the integrity of the BBB. • TEER measurements can be performed in real-time and based on measuring ohmic resistance or impedance. • The method can be applied to monitor live cells	• When reporting TEER values, laboratories should identify and report variables and the exact conditions under which TEER measurements were performed (the accuracy of the measurement techniques based on selection and usage of electrodes, temperature during measurement, medium formulation, cell culture period and passage number of cells used).	[156]
Permeability measurement	Fluorescently labelled small molecules or small radiolabelled molecules	Tightness of BBB	• Quantitative concentration measurement • To quantify the paracellular permeability • TJ barrier formation • To study the transport of tracer substances and drugs • Apparent Permeability of test compounds in either the apical or basal direction • Change in Apparent Permeability of BBB due to effect of a drug compound or pathogen	• Is typically performed at a single type of point of an experiment • Need for repeated sampling of luminal and brain side fluids. • Invasiveness.	[161, 184] [185]
Cell viability	CFDA staining	Monolayer integrity	• Quantitative analysis of viable cells • Advantages depend on which assay is used.	• Limitation depends on which assay is used.	[186]
Cellular morphology	Microscopic analysis	Monolayer integrity and BBB function	• Non-staining observation method • Live cells • Non-invasive • Simple and rapid • Observe morphological characteristics, typical normal and abnormal morphology • Provide general information on the size, shape, and growth of cells • Morphological cell analysis; quantitative characterization of cell morphology.	• Unable to provide comprehensive information about the dynamic morphology.	[163]
CFDA/CMO staining	CFDA and CMO staining	Monolayer integrity	• Detect continuous membrane contacts in confluent cultures and the intercellular gaps. • Measures enzymatic activity and cell-membrane integrity. • Simple Long-term retention	• Minimal cytotoxicity.	[187]
Cell confluence	Microscopic observation, immunostaining & TEER measurement	Integrity of BBB	• One of the essential characteristics of an *in vitro* model for endothelial permeability. • Microfilament is formed as endothelial cells grow to confluence. The DPB only appears once the cultures have reached confluence. • Ability of ECs to form and maintain the EC monolayer. • Affect cell morphology. • AJ organisation varies at various stages of cell confluence. • Control permeability.	-	[160, 161] [188, 189]
Continuity of membrane contacts	Assessing using a plasma membrane stain, CMO	Monolayer integrity	-	-	[186]
BBB cells markers expression	Immunostaining & Western blot	Characterization of BBB cells and BBB function	Immunostaining • Observe the subcellular localization and expression of AJ, TJ, ECM, and cell surface markers. • Detect multiple targets. • Qualitative and quantitative analysis of expression of cell proteins. • It also depends on the types of immunostaining used (IF (direct or indirect), ICC (direct or indirect). Western blot • Qualitative and quantitative expression of AJ, TJ, and ECM proteins. • It also depends on the types of detection the system (colorimetric, chemiluminescent, or fluorescent) and direct or indirect method used Electron microscopy • Observe minute structural alterations, surface structural alterations, and internal structural alterations. • Observe TJ formation. • Check for the formation of monolayer or multilayer. • It also depends on the types of electron microscopy used (TEM, SEM, and immunoelectron microscopy).	Immunostaining • Fixing cells (nor live cells). • Non-specific binding. • Require antibody specificity and validation. • It also depends on the types of immunostaining used. Western blot • Require specialized detection and imaging systems. • Contaminations cause high background signals. • Require antibody specificity and validation. • It also depends on the types of detection and direct or indirect method used. Electron microscopy • Expensive • Inability to analyse live cells. • It also depends on the types of electron microscopy used.	[184]
Tight junction proteins expression	Immunostaining & Western blot (mRNA and protein expression - localization), electron microscopy	Tightness of BBB			[184, 124]
Efflux transporters	mRNA and protein expression	Transendothelial transport	Gene expression • Not limited to known characteristics; permits evaluation of a large “discovery set”. • Objective and quantitative interpretation. • Effects of preanalytical variability can be assessed with RNA quality. • It also depends on the types of assays used.	Gene expression • Potential contamination. • Technically challenging to apply to fixed tissues. • Preanalytical variability (RNA quality). • It also depends on the types of assays used.	[190, 191]
SLC transporter	mRNA and protein expression	Transendothelial transport			[192, 193]
Receptor systems	mRNA and protein expression	Receptor-mediated transport			[194]
Responsiveness to regulation induced by NVU cells	Astrocytes induction: Regulation of TEER, P-gp expression and cell morphology Pericytes induction: Regulation of TEER, proteins involved in vesicular transport	Cell regulation and NVU signalling	-	-	[34, 56, 195]
Immune cell transmigration	The transmigration of calcein-labelled PBMCs	The barrier for immune cells	-	-	[155]

**Abbreviations:** CFDA, Carboxyfluorescein diacetate; CMO, CellMask Orange; PBMCs, peripheral blood mononuclear cells; DPB, dense peripheral band; IF, immunofluorescence; ICC, immunocytochemistry; TEM, transmission electron microscopy; SEM, scanning electron microscopy. DPB, dense peripheral band; IF, immunofluorescence; ICC, immunocytochemistry; TEM, transmission electron microscopy; SEM, scanning electron microscopy.

**Table 6:** Applications of *in vitro* blood-brain barrier model in neuroinfectious diseases studies.

**Infectious Agents/** ** Inflammatory Markers**	**Route**	**Mechanism**	**Impact on the BBB**	**Model**	**Disease**	**Key ** **Effector(s) (Correlation)**	**References**
Meningitic *E. coli*	NA	• Functional involvement of PDGF-BB and ICAM-1 in meningitic *E. coli* invasion of the BBB. • SphK2-S1P-S1P2-EGFR-c-Src signalling cascade. • PCN033 induction of VEGFA and Snail-1 activation of TLR2-MAPK-ERK1/2 signalling.	• Induce production and secretion of PDGF-BB enhance permeability by: -Decrease TEER. -Downregulating TJP expression (ZO-1, occludin, claudin-5). -Increase ICAM-1 expression. • PCN033 increased the BBB permeability *via*: -Downregulating and disrupting the TJP (ZO-1, β-catenin, occludin, claudin-5). -Upregulation of VEGFA. -Induction of snail-1 negatively regulates the TJ protein expression.	*In vitro* hBMEC model	Bacterial meningitis	The bacteria and proinflammatory cytokines/ chemokines	[105, 207, 208]
*S. pneumoniae*	NA	• LR-pneumococcal CbpA interaction	• No significant disruption of endothelial integrity.	Mouse and human BMECs *in vitro* model	Pneumococcal meningitis	NA	[209, 210]
Group B *Streptococcus*	NA	• SfbA-fibronectin-integrin interaction	• Disruption of the tight junction proteins.	hBMEC *in vitro*, iPSC-derived BMEC model	Streptococcal meningitis	NA	[211, 212]
SARS-CoV-2	Endocytosis? Trojan horse? Intracellular transmigration	• Spike protein-ACE2 interactions	• Alter the BBB integrity o Reduce TEER o Increase passive paracellular permeability o Decreased ZO-1 localisation to cell-cell junctions. o Structural reconfiguration. o Decrease TJ proteins expression: ZO-2, Claudin-5, JAM-2. • Increased ECM enzymes (MMP3. • Increased cell adhesion molecules (VCAM-1 and ICAM-1). • Increased endothelial pro-inflammatory response (CCL5, CXCL10).	*In vitro* hBMEC model, 2D & 3D human BBB model	COVID-19-associated neurological disease	The virus and structural reconfiguration of TJ proteins, proinflammatory cytokines/ chemokines	[224, 226]
HIV-1	NA	NA	• Reduced expression of Cx43, a-SMA), α1-integrin, and PDGF-Rβ). • Increase in inflammatory factor secretion. • Adhesion molecules upregulation. • Development of a migratory phenotype. • Disruption of BBB. • Downregulation of fibronectin and nidogen in pericytes. • Reduction in TIMP3.	hBMVEC-brain pericytes co-culture model with monocyte adhesion/ migration	HIV-1-associated neurocognitive disorder	NA	[235]
Semliki forest virus (SFV)	Transcellular	Lysine facilitated viruses with the most significant reliance on binding to GAGs more efficiently crossed an *in vitro* BBB.	Significant reduction in TEER	*In vitro* human BBB	Virus encephalitis	The ability to replicate in the brain	[214]
Japanese encephalitis virus (JEV)	Passive diffusion	JEV is able to infect endothelial cells, release pro-inflammatory markers from these cells, increased permeability of BBB, infect astrocytes, release pro-inflammatory markers.	Drop in TEER	*In vitro* human BBB	Encephalitis	Inflammatory cytokines/ chemokines from HBECs and astrocytes	[13]
Dengue virus (DENV)	Transcellular, paracellular, ‘Trojan horse’	NA	• Decrease TEER. • Increase permeability. • Subcellular localization of ZO-1 and claudin-1. • Transmigration of monocytes/macrophages.	*In vitro* MBECs model *in vitro* MBECs-astrocytes model	DENV infection	The virus and inflammatory cytokines/ chemokines	[239]
Zika virus (ZIKV)	Transcellular	NA	Without compromising TEER and paracellular permeability or the viability of the i-BEC monolayer.	The human iPSC-derived i-BEC, i-NPs and i-Ns BBB model	ZIKA infection	The virus and AXL-mediated ZIKV entry	[186]
HCV	receptor-dependent entry pathway	HCVpp and VSV-Gpp receptors hCMEC/D3 and HBMEC interaction	Disrupt endothelial cell integrity o Increased paracellular permeability.	hCMEC/D3 and HBMEC *in vitro* BBB model	HCV infection-associated neuropathology	Direct infection	[245]
African trypanosome	Transmigration	NA	NA	ECV304-C6 BBB model	African trypanosome infection	Increase of the trypanosomal inoculum	[246]
*P. berghei* *P. falciparum*	Cross-presentation of PbA, cytoadherence	hICAM-1 of hCMEC/D3 cells and a trypsin-sensitive antigen of *P. falciparum* RAOL cytoadherence	• Disorganization of ZO1 • Increased endothelial cell permeability.	*In vitro* MBECs PbA cross-presentation model, co-culture of *P. falciparum* RAOL-PRBCs and hCMEC/D3 cells model	CM	NA	[247, 24]
*Taenia solium* *T. gondii*	Leukocyte transendothelial migration pathway, ‘Trojan horse’	NA	• Overexpressed of VEGF-A in astrocytes. • BBB disruption • -Decreased immunoreactivity to EBA marker. • -Increased permeability for IgG. • Upregulation of ICAM-1. • Increased IL-6 and MCP-1.	*In vitro* HUVEC model, rat brain endothelial and glial cells *in vitro* BBB model.	Neurocysticercosis	Parasite itself	[108, 249]

**Abbreviations:** NA, not available; PDGF-BB, platelet-derived growth factor-BB; ICAM-1 intracellular adhesion molecule-1; VEGFA, vascular endothelial growth factor A; ZO-1, zonula occludens-1; TJ, tight junction; hBMEC, human brain microvascular endothelial cell; GAGs, glycosaminoglycans; HBECs, human brain endothelial cells; i-BEC, induced-brain endothelial cells; iPSC, induced-pluripotent stem cells; i-NPs, induced-neural progenitor cells; i-Ns, induced-mature neurons; αSMA, alpha smooth muscle actin; PDGF-Rβ, platelet-derived growth factor-B receptor; VCAM, vascular cell adhesion molecule; HUVEC, human umbilical vein endothelial cell.

## LIST OF ABBREVIATIONS

BBB: Blood-brain Barrier
CNS: Central Nervous System
EBA: Endothelial Barrier Antigen
eNOS: Endothelial Nitric Oxide Synthase
FGF: Fibroblast Growth Factor
GDNF: Glial-derived Neurotrophic Factor
LPS: Lipopolysaccharide
MIP2: Macrophage-inflammatory Protein 2
NO: Nitric Oxide
SLC: Solute-like carrier
TEER: Transendothelial Electrical Resistance
TJP: Tight Junction Proteins
